# CyTOF mass cytometry reveals phenotypically distinct human blood neutrophil populations differentially correlated with melanoma stage

**DOI:** 10.1136/jitc-2019-000473

**Published:** 2020-09-09

**Authors:** Yanfang Peipei Zhu, Tobias Eggert, Daniel J Araujo, Pandurangan Vijayanand, Christian Hermann Ottensmeier, Catherine C Hedrick

**Affiliations:** 1 Inflammation Biology, La Jolla Institute for Immunology, La Jolla, California, USA; 2 Division of Vaccine Discovery, La Jolla Institute for Immunology, La Jolla, California, USA; 3 School of Cancer Sciences, University of Southampton Faculty of Medicine, Southampton, UK

**Keywords:** haematology, immunology

## Abstract

**Background:**

Understanding neutrophil heterogeneity and its relationship to disease progression has become a recent focus of cancer research. Indeed, several studies have identified neutrophil subpopulations associated with protumoral or antitumoral functions. However, this work has been hindered by a lack of widely accepted markers with which to define neutrophil subpopulations.

**Methods:**

To identify markers of neutrophil heterogeneity in cancer, we used single-cell cytometry by time-of-flight (CyTOF) coupled with high-dimensional analysis on blood samples from treatment-naïve patients with melanoma.

**Results:**

Our efforts allowed us to identify seven blood neutrophil clusters, including two previously identified individual populations. Interrogation of these neutrophil subpopulations revealed a positive trend between specific clusters and disease stage. Finally, we recapitulated these seven blood neutrophil populations via flow cytometry and found that they exhibited diverse capacities for phagocytosis and reactive oxygen species production in vitro.

**Conclusions:**

Our data provide a refined consensus on neutrophil heterogeneity markers, enabling a prospective functional evaluation in patients with solid tumors.

## Introduction

Neutrophils are bone marrow (BM)-derived myeloid cells that play pivotal roles in anticancer immunity.^1^ Neutrophils are produced at a rate of 10^11^ per day^2 3^ and comprise 50%–70% of blood leukocytes. Due to this rapid turnover in the body, neutrophils have traditionally been viewed as a homogeneous population. However, recent work has shown that they exhibit a longer life cycle than previously thought,^4^ reviving interest in the possibility of distinct neutrophil populations.^3 5^

Spurred on by such findings, several groups have since identified and characterized several neutrophil subpopulations. For example, use of density gradient separation has uncovered low-density neutrophil (LDN) and high-density neutrophil (HDN) populations, each with opposing actions in immune regulation and cancer progression.^6^ Marini and coauthors employed flow cytometry to show that CD10^+^ and CD10^−^ neutrophils represent populations with opposing effects on T-cell proliferation.^7^ Pillay and colleagues identified three neutrophil subpopulations, based on their differential expression of CD16 and CD62L, with each exhibiting specific maturation and activation statuses.^8^ CD45RA, CD63, and CD11b also indicate activation statuses in certain neutrophil subsets.^9 10^ Singhal and collaborators isolated a CD14^+^ neutrophil subpopulation with antitumor functions, including enhancement of effector T cell-based production of interferon-g and granzyme B.^11^ Evrard and colleagues have demonstrated a CD15^+^CD49^+^CD101^−^ neutrophil precursor (preNeu).^12^ Our group has identified a CD117^+^CD66b^+^CD38^+^ human neutrophil progenitor (hNeP), which was also found in the blood of tumor-bearing animals.^13^ Additional work in this area is summarized in two excellent review articles.^14 15^ Nevertheless, a lack of widely accepted subpopulation markers has hindered our understanding of neutrophil heterogeneity. Indeed, the neutrophil subpopulations thus far described likely represent intersecting populations. For example, flow cytometry analysis suggests that CD10^+^ and CD10^−^ neutrophil subpopulations are fractionated into both LDN and HDN layers.^7^ Furthermore, the CD10^+^ expression demonstrated by Marini *et al*^7^ is shared by the CD16^bright^ subpopulation reported by Pillay *et al*.^8^ Specific CD14^+^ neutrophils present a CD10^−^ phenotype,^11^ suggesting this subpopulation overlaps with the CD10^−^ neutrophil population.^7^ The CD14^+^ neutrophil subpopulation also present a CD49d^+^ phenotype, indicating that it overlaps with the CD49d^+^ preNeu demonstrated by Evrard *et al*,^12^ as well as a CD49d^+^CD62L^lo^ neutrophil subpopulation (‘aged neutrophil’) reported by Casanova-Acebes *et al*.^16^ To determine the extent to which previously reported neutrophil subpopulations intersect, high-dimensional analysis of neutrophil heterogeneity on a single-cell basis is imperative.

We and others have employed high-dimensional approaches such as single-cell cytometry by time-of-flight (CyTOF) and single-cell RNA sequencing (scRNA-seq) to address neutrophil heterogeneity. These endeavors demonstrate that the neutrophil lineage comprises a heterogeneous pool in mouse and human BM.^12 13^ Additionally, scRNA-seq analyses reveal six neutrophil clusters with distinct transcriptional signatures in human lung tumors, but the surface markers needed to classify these populations were not identified.^17^ Interestingly, work in this field has also suggested differential involvement of neutrophil subpopulations in cancer.^18 19^ Thus, the development of consensus neutrophil markers is required for improving our understanding of neutrophil biology and its relationship to disease progression.

Here, we use a CyTOF panel of the most commonly used surface markers of neutrophil maturation, activation, and function to comprehensively investigate neutrophil heterogeneity in whole blood (WB) from treatment-naïve patients with melanoma. High-dimensional analysis of this dataset revealed seven neutrophil subpopulations associated with disease stage and which are reproducible during manual gating in flow cytometry. Finally, we found that these seven neutrophil subpopulations harbor distinctive functions, demonstrated by their differential capacities for phagocytosis and reactive oxygen species (ROS) production.

## Methods

### Melanoma patient blood collection

Blood samples from patients with melanoma who were treatment-naïve after surgical resection were collected in EDTA-coated tubes by the Biospecimen Repository Core Facility at the University of Kansas Cancer Center and delivered to La Jolla Institute for Immunology (LJI) via overnight shipping. Concurrently, blood from healthy donors was collected in EDTA-coated tubes at LJI. To ensure uniform treatment between control and experimental materials, all healthy donor blood samples were stored at 4°C overnight and then processed the next morning.

### Cell suspension from human WB

WB was subject to red blood cell (RBC) lysis (RBC lysis buffer, eBiosciences) twice at room temperature (RT) for 10 min. Cells were then washed with staining buffer (Dulbecco’s phosphate-buffered saline+1% human serum+0.1% sodium azide+2 mM EDTA) and filtered through a 70 µm strainer. Cells were pelleted by centrifugation and suspensions were prepared with gentle pipetting to reach final concentration of 3×10^6^ cells per 100 μL buffer.

### Mass cytometry antibodies

Metal-conjugated antibodies were purchased directly from Fluidigm for available targets. For all other targets, purified antibodies were purchased as described before.^20^ Antibody conjugations were prepared using the Maxpar Antibody Labeling Kit (Fluidigm) according to the manufacturer’s recommendations. Afterwards, Maxpar-conjugated antibodies were stored in phosphate-buffered saline-based antibody stabilization solution (Candor Biosciences) supplemented with 0.05% sodium azide at 4°C. All antibodies were titrated before use.

### Mass cytometry (CyTOF)

CyTOF was performed following previously described protocols.^20^ For viability staining, cells were washed in phosphate-buffered saline and stained with Cisplatin (Fluidigm) at a final concentration of 5 µM. Prior to surface staining, RBC-lysed WB cells were resuspended in staining buffer for 15 min at RT to block Fc receptors. The surface antibody cocktail listed in table 1 was added into cell suspensions for 1 hour at 4°C. The cells were then washed with staining buffer and fixed with 1.6% paraformaldehyde (Thermo Fisher) for 15 min at RT. Afterwards, 1 mL of intercalation solution for each sample was prepared by adding Cell-ID Intercalator-Ir (Fluidigm) into Maxpar Fix and Perm Buffer (Fluidigm) to a final concentration of 125 nM (a 1000× dilution of the 125 µM stock solution) and vortex to mix. After fixation, the cells were resuspended with the intercalation solution and incubated overnight at 4°C. Cells were then washed in staining buffer and then with subsequent washes in Cell Acquisition Solution (CAS) (Fluidigm) to remove buffer salts. Next, the cells were resuspended in CAS with a 1:10 dilution of EQ Four Element Calibration beads (Fluidigm) and filtered through a 35 µm nylon mesh filter cap (Corning, Falcon). Samples were analyzed on a Helios 2 CyTOF Mass Cytometer (Fluidigm) equipped with a Super Sampler (Victorian Airship & Scientific Apparatus) at an event rate ≤500 events/s. Mass cytometry data files were normalized using the bead-based Normalizer^21^ and were analyzed using Cytobank analysis software (https://www.cytobank.org/). For analysis of mass cytometry data with a self-organizing map (FlowSOM) in Cytobank, hierarchical clustering was used to determine seven metaclusters based on median marker expression (after arcsinh transformation with cofactor equal to 5) from the visualization of t-distributed stochastic neighbor embedding (viSNE) results.

**Table 1:** Cytometry by time-of-flight antibody panel

Leukocyte lineage			Maturation			Heterogeneity (function)		
Isotope	Metal	Specificity	Isotope	Metal	Specificity	Isotope	Metal	Specificity
89	Y	CD45	146	Nd	CD64	153	Eu	CD14
113	In	CD3	151	Eu	CD49d	161	Dy	Arg1
113	In	CD127	154	Sm	CD117	168	Er	CD304 (Nrp1)
115	In	CD41	156	Gd	CD10	171	Yb	HLA-A/B/C
115	In	CD235a	158	Gd	CD101	174	Yb	HLA-DR
141	Pr	CD11c	165	Ho	CD16	Heterogeneity (migration)		
143	Nd	CD123	166	Er	CD34	Isotope	Metal	Specificity
143	Nd	CD203c	167	Er	CD38	147	Sm	CD182 (CXCR2)
144	Nd	CD19	172	Yb	CD15	159	Tb	CD197 (CCR7)
148	Nd	CD11b	Heterogeneity (adhesion/activation)			175	Lu	CD184 (CXCR4)
152	Sm	CD66b	Isotope	Metal	Specificity	Proliferation		
163	Dy	CD86	142	Nd	CD11a (LFA-1)	Isotope	Metal	Specificity
164	Dy	Siglec 8	145	Nd	CD62L	127	I	IdU
169	Tm	CD33	149	Sm	CD48	162	Dy	CD71
176	Yb	CD56	155	Gd	CD45RA			
Heterogeneity (other)			170	Er	CD35			
Isotope	Metal	Specificity						
160	Gd	CD79b						

### Flow cytometry and cell sorting

All fluorescence-activated cell sorting (FACS) staining was performed in staining buffer at 4°C. Cells were filtered through sterile, 70 µm cell strainers to obtain single-cell suspensions (30 000 cells per μL for flow cytometry analysis, 0.5–2×10^7^ cells per mL for sorting). Prior to surface staining, RBC-lysed WB cells were resuspended in staining buffer to block Fc receptors for 15 min at RT. Surface staining was performed for 30 min in a final volume of 500 uL for FACS sorts and 100 μL for standard flow cytometry. Cells were washed twice in at least 200 μL FACS buffer before acquisition. FACS was performed via an Aria II and Aria-Fusion (BD Biosciences) and conventional flow cytometry via an LSRII and LSR Fortessa (BD Biosciences). All flow cytometry was performed on live cells. Percentages of CD45^+^ immune cells were calculated by forward and side scatter and viability analyses of live cells. All analyses and sorts were repeated at least three times, and the purity of each sorted fraction was determined visually and by FACS reanalysis of surface markers. Data were analyzed using FlowJo software V.10.5.

### Phagocytosis

Phagocytic capacities of neutrophils were assessed with a Phagocytosis Assay Kit (Red Zymosan) (Abcam) following the manufacturer's protocol. RBC-lysed WB cells were diluted to a concentration of 3×10^6^ cells in 1 mL buffer and incubated with 5 µL of zymosan slurry per sample at 37°C and 5% CO_2_ for 2.5 hours. Afterwards, the samples were centrifuged for 5 min at 400×*g* and then stained with 100 µL antibody cocktail for flow cytometry-based detection of phagocytosis of red zymosan particles by neutrophils.

### Cellular ROS detection

ROS production by neutrophils was detected with a Cellular ROS Detection Assay Kit (Abcam) following the manufacturer's manual. The RBC-lysed WB cells were diluted to the concentration of 3×10^5^ cells in 100 mL buffer. Pretreatment with ROS inhibitor (N-acetyl-L-cysteine) was carried out for the negative control group at 37°C, 5% CO_2_ for 30 min. ROS detection antibody then added into the antibody cocktail for flow cytometry-based detection of neutrophil ROS production. ROS inducer (pyocyanin) was added to all groups and incubated for 30 min prior to acquisition on the cytometer.

### Quantification and statistical analyses

**Figure 1:**
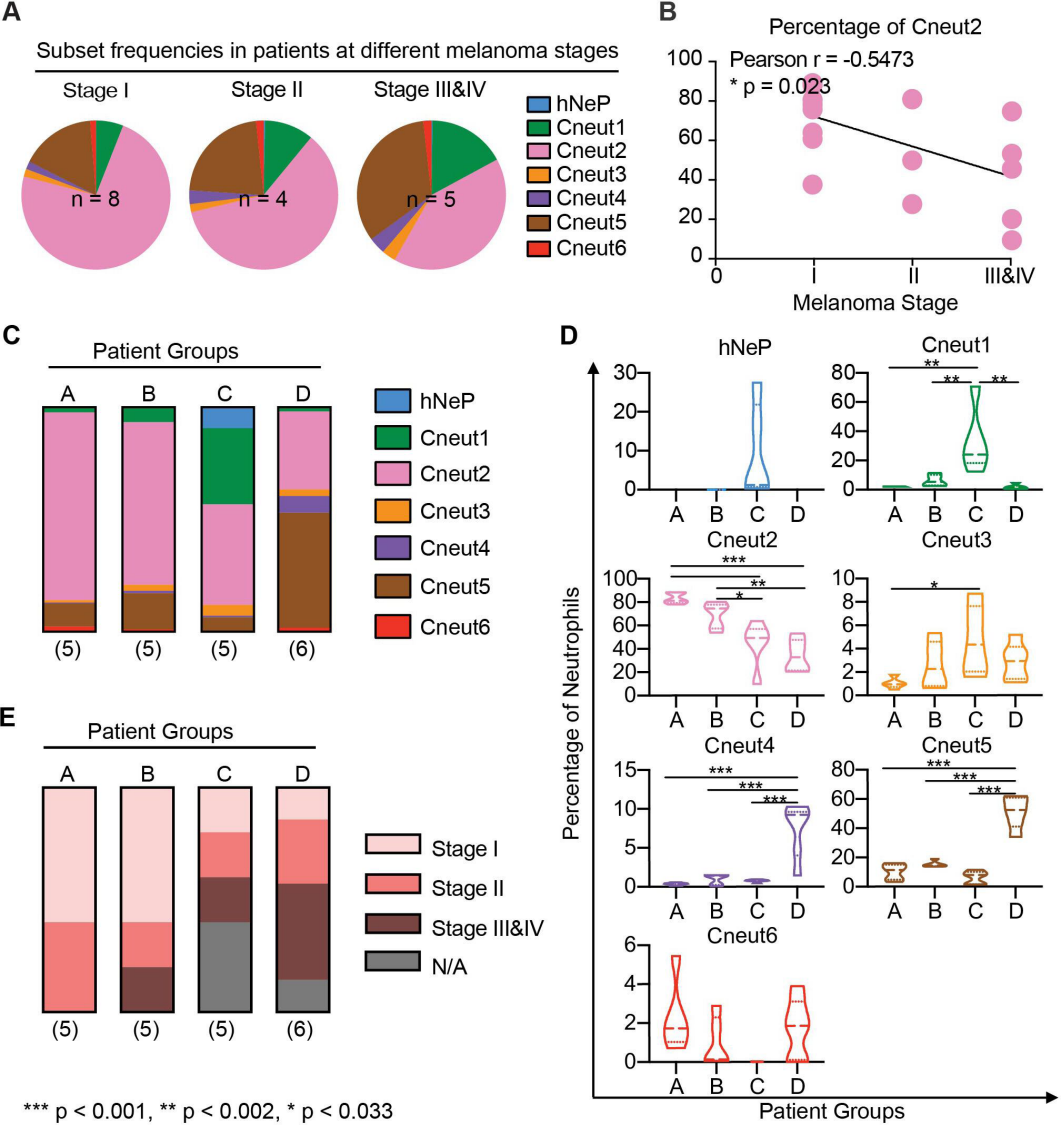
Neutrophil heterogeneity in patients with melanoma correlates with disease stage. (A) Pie charts show mean percentages for each FlowSOM cluster (hNeP and Cneut1–6) in total blood neutrophils of patients grouped by melanoma stage. Only patients with a melanoma stage diagnosis shown in table 2 were used for this analysis. The numbers of subjects in each melanoma stage are indicated on the graph. (B) Line regression analysis shown in dot plot depicting correlations between neutrophil cluster frequency and melanoma stage. Each dot represents one patient. Pearson analysis results are shown for each cluster. P values were calculated based on two-tailed comparisons with 95% CIs and shown in APA style. (C) Bar graph shows the mean percentage of each cluster in patient groups A–D. All patients were used for this analysis, regardless of whether or not they received a melanoma stage diagnosis (table 2). The numbers of subjects in each group are indicated below each column. (D) Violin plots show the neutrophil cluster frequency in patient pools A–D. Quartiles and median values of each patient pool are indicated as dotted lines. All patients were used for this analysis regardless of whether or not they received a melanoma stage diagnosis (table 2). Differences between groups were determined by using ordinary one-way analysis of variance and Tukey’s multiple comparison test with a single pooled variance. Error bars indicate mean±SD. P values were calculated based on two-tailed comparisons with 95% CIs and shown in APA style. (E) Bar graph shows the percentage of patients at different melanoma stages (table 2) in patient groups –D. All the patients were used for this analysis regardless of whether or not they received a melanoma stage diagnosis (table 2). The numbers of patients in each pool are indicated below each column. APA' American Psychological Association; FlowSOM, analysis of mass cytometry data with a self-organizing map; hNeP, human neutrophil progenitor; N/A, missing diagnosis information of patients.

**Figure 2:**
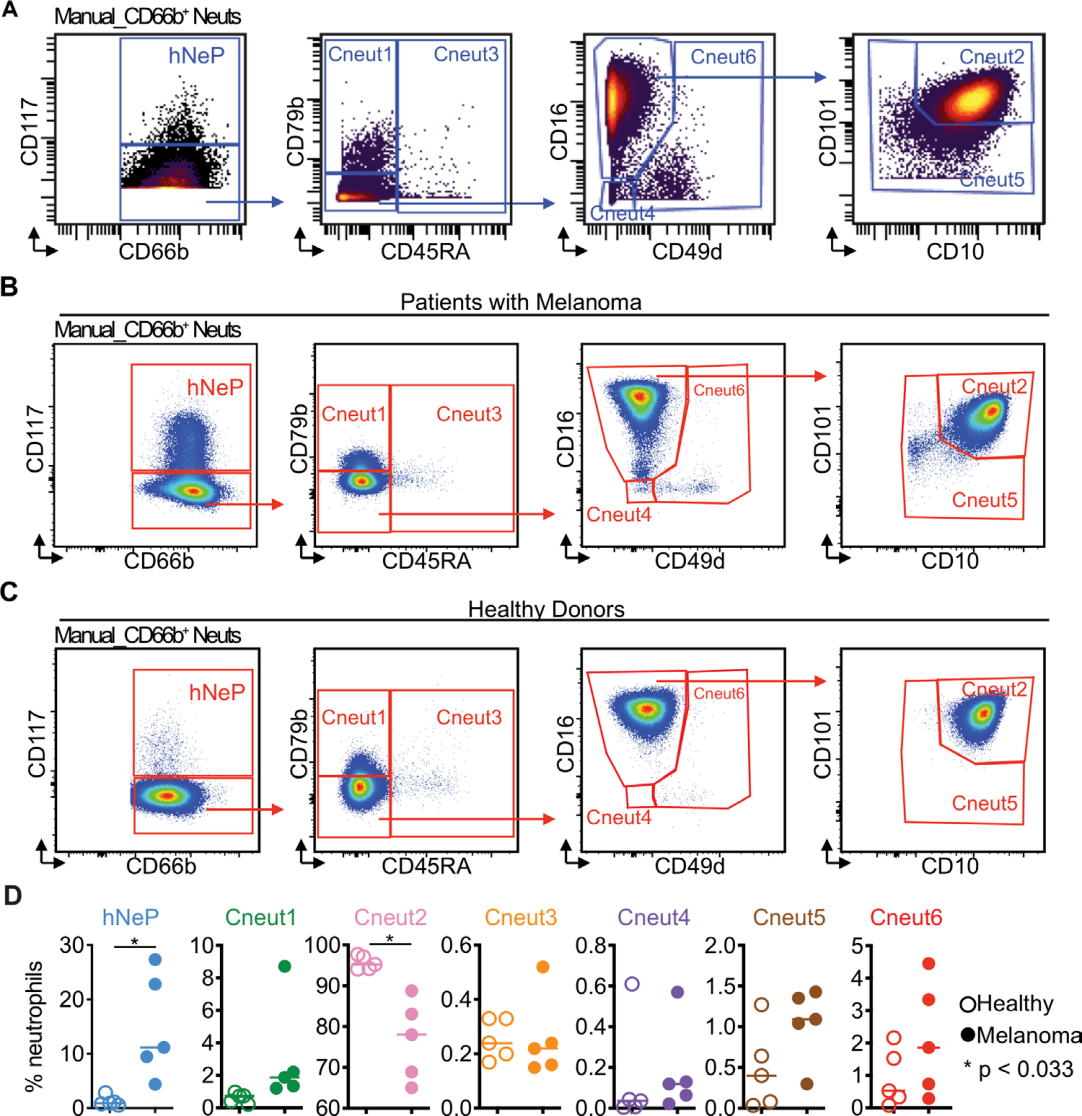
Flow cytometry replicates the seven neutrophil subpopulations. (A) CD66b^+^ blood neutrophils were manually selected and subjected to sequential gating to identify the neutrophil subpopulations with CyTOF. Scales are shown in arcsinh transformation with cofactor equal to 5. (B) The gating strategy from (A) was validated by flow cytometry in treatment-naïve patients with melanoma. Scales are shown in biexponential scale. (C) Flow cytometry analysis of healthy donor’s blood neutrophils with the gating strategy from (A). Scales are shown in biexponential scale. (D) Flow cytometry analysis of the frequency of each manually gated neutrophil subpopulation in total blood neutrophils. Five healthy donors (age 23–46, two women and three men) and five treatment-naïve patients with melanoma (aged 59–79 years, two women and three men) were analyzed. Each dot represents the result of one patient. Paired t-tests were used to compare the differences between healthy patients and patients with melanoma. Error bars indicate mean±SD. P values were calculated based on two-tailed comparisons with 95% CIs and shown in APA style. CyTOF, cytometry by time-of-flight; hNeP, human neutrophil progenitor.

**Figure 3:**
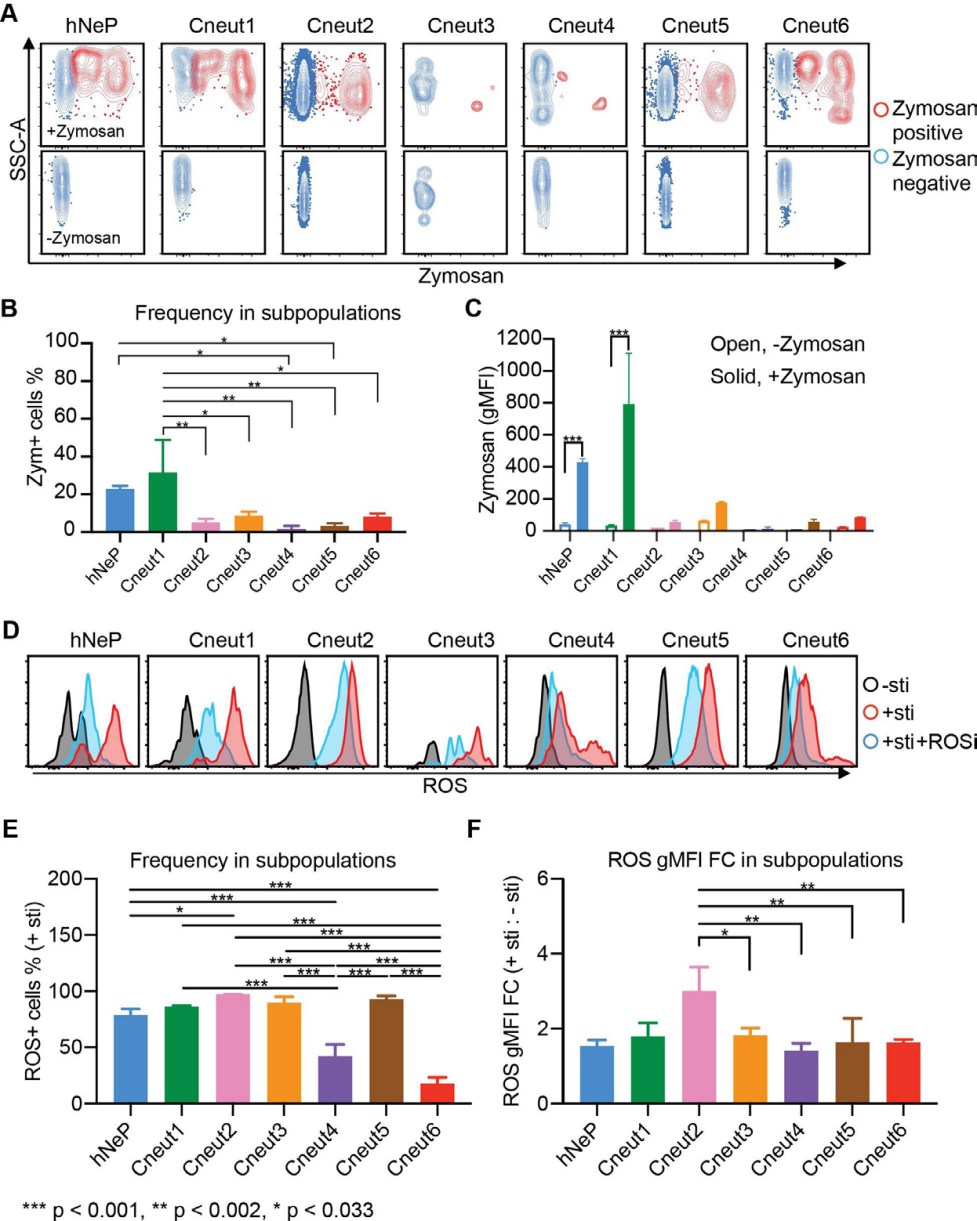
Seven neutrophil subpopulations harbor diverse phagocytic and ROS-producing capacities. Three randomly selected melanoma-naïve patients (ages 59, 77 and 79; one woman and two men) were analyzed with flow cytometry. (A–C) RBC-lysed blood samples were incubated with prelabeled zymosan particles for 2.5 hours. Afterwards, the cells were harvested and stained with the flow cytometry panels described in figure 2 and online supplementary figure 6C. Each neutrophil subpopulation was gated to evaluate its uptake of zymosan particles. (A) The zymosan-positive cells are shown in red; the zymosan-negative cells are shown in blue. The no-zymosan group (−zymosan) is shown in the bottom panels as the control group. (B) Percentage of the zymosan-positive cells (red dots in A) in each gated neutrophil subpopulation. Error bars indicate mean with SD. (C) gMFI of zymosan in each gated neutrophil subpopulation. Error bars indicate mean with SD. (D) Seven neutrophil subpopulations’ ability to produce ROS is determined by flow cytometry. RBC-lysed blood samples were split into three groups and incubated with −sti, +sti, or sti+ROSi. Afterwards, each neutrophil subpopulation was gated for evaluation of ROS+ cells. Histogram plots show the gated neutrophil subpopulations in each group: −sti is shown in black; +sti is shown in red; +sti+ROSi is shown in blue. (E) Percentage of the ROS+ cells in each gated neutrophil subpopulation from the +sti group. Error bars indicate mean with SD. (F) ROS gMFI FC of each gated neutrophil subpopulation from the +sti group to the −sti group. Statistics: (B, E, F) differences between groups were determined by using ordinary one-way ANOVA and Tukey’s multiple comparison test with a single pooled variance. (C) Ordinary two-way ANOVA and Sidak’s multiple comparison test with individual variances computed for each comparison were performed to compare between +zymosan and −zymosan groups. Error bars indicate mean±SD. P values were calculated based on two-tailed comparisons with 95% CIs and shown in APA style. APA, American Psychological Association; ANOVA, analysis of variance; FC, fold change; gMFI, geometric mean fluorescence intensity; hNeP, human neutrophil progenitor; RBC, red blood cell; ROS, reactive oxygen species; ROSi, reactive oxygen species inhibitor; SSC-A, −sti, no stimulation; +sti, ROS inducer (pyocyanin) alone.

Data for all experiments were analyzed with Prism software (GraphPad). For figure 1, linear regression and Pearson correlation coefficients were used to determine the correlation between neutrophil cluster frequencies with melanoma stages. Differences between groups were determined via ordinary one-way analysis of variance (ANOVA) and Tukey’s multiple comparison tests with a single pooled variance. For figure 2, paired Student’s t-tests were used to compare the differences between healthy patients and patients with melanoma. For figure 3B and E, F and online supplementary figure 7A, C–E, differences between groups were determined by using ordinary one-way ANOVA and Tukey’s multiple comparison tests with a single pooled variance. For figure 3C and online supplementary figure 7B and F, ordinary two-way ANOVA and Sidak’s multiple comparisons tests with individual variances computed for each comparison were performed to compare the +zymosan and −zymosan groups. Error bars indicate means±SD. P values were calculated by two-tailed comparisons with 95% CIs and shown in American Psychological Association (APA) style.

10.1136/jitc-2019-000473.supp1 Supplementary data

**Table 2:** Patient demographics and tumor characteristics

Characteristics		
Age (years)		
	Median (range)	69 (24–82)
Sex, n (%)		
	Male	12 (57.1)
	Female	9 (42.9)
Primary tumor thickness (mm)		
	n (range)	13 (0.4–16)
	0–1.00, n (%)	4 (19)
	1.00–4.00, n (%)	6 (28.6)
	>4.00, n (%)	3 (14.3)
	Missing, n (%)	8 (38.1)
Primary tumor ulceration status, n (%)		
	Absent	9 (42.9)
	Present	4 (19)
	Missing	8 (38.1)
Primary tumor anatomical site, n (%)		
	Arms/legs	5 (23.8)
	Torso	5 (23.8)
	Head/neck	7 (33.3)
	Missing	4 (19)
Primary tumor mitosis, n (%)		
	Absent	5 (23.8)
	Present	7 (33.3)
	Missing	9 (42.9)
AJCC Cancer Staging Manual stage at pathological diagnosis, n (%)		
	Stage I	8 (38.1)
	Stage II	4 (19)
	Stage III/IV	5 (23.8)
	Missing	4 (19)

AJCC, American Joint Committee on Cancer.

## Results

### CyTOF reveals seven neutrophil clusters in blood from patients with melanoma

In order to identify novel blood neutrophil populations, we used CyTOF mass cytometry to analyze RBC-lysed, fresh blood samples from a cohort of 21 patients with melanoma (table 2). At the time of sample collection, these patients were recently diagnosed, had not received any treatment for their condition (treatment-naïve), and ranged from 24 to 82 years of age, with a median age of 69 years (table 2). After processing, samples were subjected to a CyTOF antibody panel that simultaneously measured the expression of 40 neutrophil surface markers (table 1). Leukocyte lineage markers (table 1) were used to perform viSNE-automated analysis to study live CD45^+^ single cells in blood (online supplementary figure 1A). Neutrophils (CD66b^+^ cell-enriched cluster) were distinguishable from all other leukocytes using this strategy (online supplementary figure 1B).

**Figure 4:**
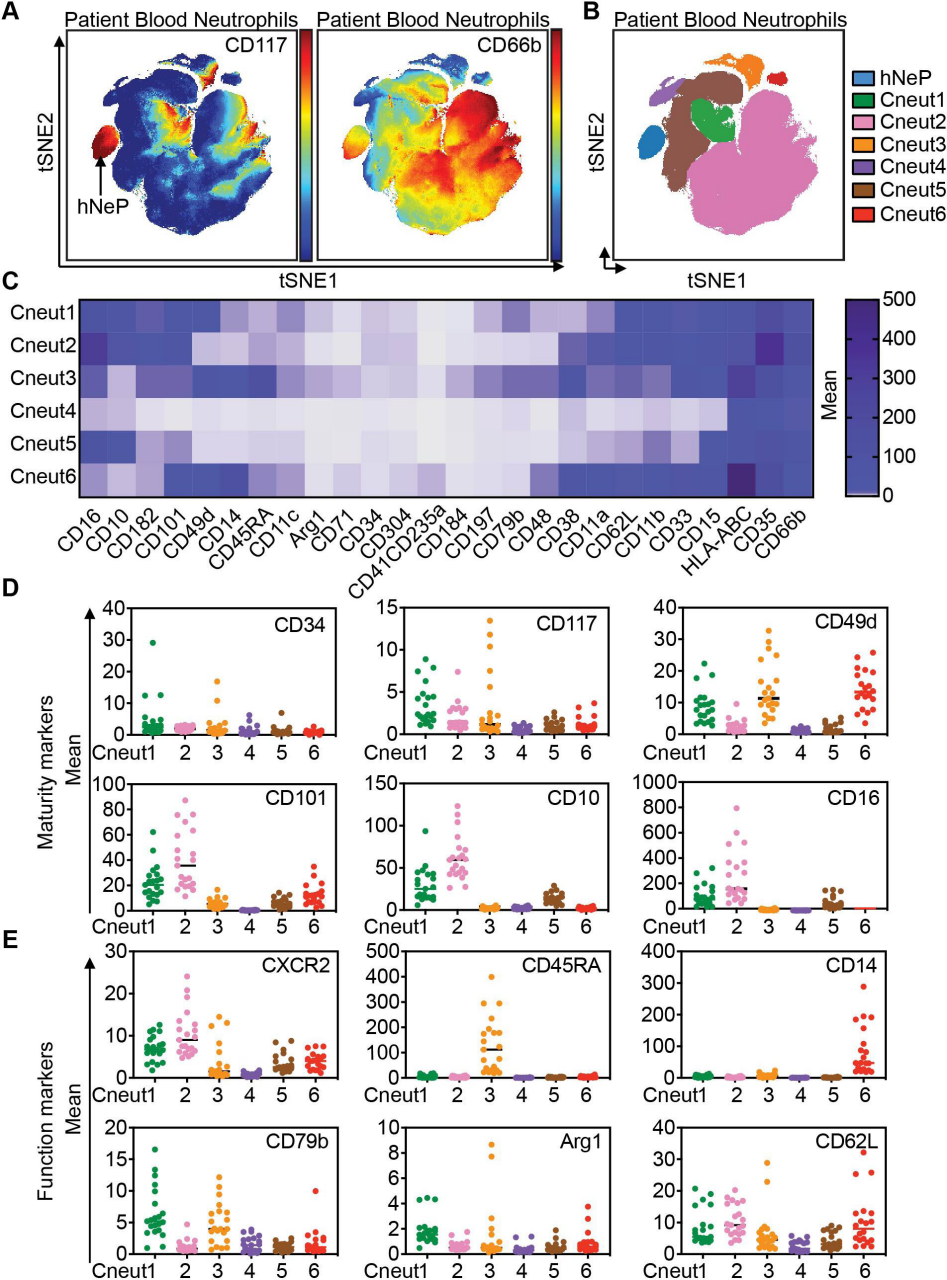
CyTOF-based analysis of blood from patients with melanoma reveals seven automated neutrophil clusters. The blood neutrophils (the CD66b^+^ automated cluster from online supplementary figure 1B) from treatment-naïve patients with melanoma were subjected to automated analysis. (A) Mean intensities of CD117 and CD66b expression are shown on viSNE map as spectrum colored dots (low in blue, high in red). hNeP was identified on the viSNE map based on the expression of CD117^+^CD66b^+^. (B) FlowSOM analysis of the viSNE results revealed seven automated clusters. (C) Heatmap shows the mean intensity of each marker in the six unidentified automated clusters on a global scale. (D) Dot plot shows the mean intensity of each maturity marker in the six unidentified automated clusters. Each dot represents the result of one patient. (E) Dot plot shows the mean intensity of each functional marker in the six unidentified automated clusters. Each dot represents the result of one patient. CyTOF, cytometry by time-of-flight; FlowSOM, analysis of mass cytometry data with a self-organizing map; hNeP, human neutrophil progenitor; viSNE, visualization of t-distributed stochastic neighbor embedding.

We focused on the CD66b^+^ cell-enriched cluster to analyze the heterogeneity of neutrophils across all patient samples. Our results demonstrate that these samples contained a CD117^+^CD66b^+^, hNeP population (figure 4A), which we have formerly identified.^13^ In addition, FlowSOM analyses^22^ revealed another six neutrophil clusters (termed Cneut1 through Cneut6, figure 4B), each with distinct surface marker profiles (figure 4C). Two randomly selected healthy donors’ blood were used as a control group to identify bona fide blood neutrophil populations (online supplementary figure 2A). Compared with healthy donor blood neutrophils, neutrophils from patients with melanoma displayed higher heterogeneity and the frequency of the largest neutrophil cluster (Cneut2) decreased from >95% in healthy donors to <90% in patients with melanoma (online supplementary figure 2B). The marker profile of Cneut2 in healthy blood was not significantly different from Cneut2 in patients with melanoma (online supplementary figure 2C). As maturity markers are commonly used to distinguish circulating neutrophil subpopulations in cancer,^12 23^ we next quantified expression of these markers in the Cneut1-Cneut6-clustered neutrophils. We found that cluster Cneut2 expresses high levels of CD101, CD10, and CD16 compared with the other clusters, indicating its enrichment for terminally differentiated, mature neutrophils (figure 4C, D).^7^ Furthermore, CD10^lo^ clusters (Cneut1 and Cneut3-Cneut6) express reduced levels of CD101, highlighting their immaturity^12^ when compared with the Cneut2 cluster (figure 4D). The Cneut1, Cneut3, and Cneut6-clustered neutrophils express progenitor markers (CD34 and CD117) at a lower level than hNeP, but at a higher level than other clusters (figure 4D and online supplementary figure 3A). In the Cneut5 cluster, expression of CD34 and CD117 was diminished, compared with Cneut1, Cneut3, and Cneut6 (online supplementary figure 3A). In contrast, CD10 and CD16 levels were higher in the Cneut5 cluster, compared with Cneut3 and Cneut6, intimating that this cluster denotes immature neutrophils (figure 4D). Moreover, Cneut3 and Cneut6 both express CD101^−^CD49d^+^, which suggests these clusters belong to the preNeu population.^12^

We further characterized these clusters by comparing functional markers previously used to evaluate neutrophil populations (figure 4E). In agreement with the initial findings in figure 4C, Cneut2 expressed the highest CXCR2 levels, confirming its maturation status. Interestingly, Cneut3 and Cneut6, which are both identified as belonging to the CD101^−^CD49d^+^ preNeu pool, exhibited differential levels of CD45RA and CD14. CD45RA was expressed only by Cneut3, whereas CD14 was exclusively expressed by Cneut6. Additionally, Cneut3 exhibited higher CXCR4 levels, compared with cluster Cneut6, which suggests Cneut3 represents a previously reported senescent (CXCR4^+^CD49d^+^CD62L^lo^) neutrophil population (online supplementary figure 3B).^16^ Finally, Cneut1 and Cneut6 expressed low amounts of CD16, compared with Cneut2 (figure 4D), and stained positive for CD62L (figure 4E), suggesting these cells are the CD16^dim^CD62L^bright^ band cells that have been described previously.^8^ Overall, our approach was able to recapitulate both novel and established neutrophil populations from the peripheral blood of patients with melanoma.

### Neutrophil heterogeneity correlates with melanoma stage

Based on the aforementioned results, we sought to determine the frequency of each neutrophil cluster in our patient samples. We did not observe enrichment of specific neutrophil clusters related to patient demographics, such as age and sex, or tumor characteristics, such as the anatomical site or ulceration status. No significant correlations were observed between cluster frequency changes and these patient demographics described in table 2. However, the overall frequency of precursors/immature neutrophils (hNeP, Cneut1, and Cneut3-Cneut5) increased up to fourfold with disease prognosis (figure 1A and online supplementary figure 4A, B), whereas positive correlation was observed between Cneut2’s percentage of total neutrophils with melanoma stage (Pearson r=0.5473, p=0.023) determined by regression analysis (figure 1B). Thus, we hypothesized that different patterns of neutrophil heterogeneity demonstrated by cluster frequencies are predictive of disease stage. To test this hypothesis, we categorized patients into four different groups (A–D) based on these patterns, as analyzed by viSNE (figure 1C and online supplementary figures 4C, D). From this analysis, we determined that patients in group C have the highest hNeP, Cneut1 and Cneut3 frequencies. Patients included in group D have the highest Cneut4 and Cneut5 frequencies (figure 1D). The frequency of Cneut2 progressively decreases from patient groups A–D. We next investigated the melanoma stage of the patients in these four groups (figure 1E). Interestingly, patients in group A were all diagnosed with early-stage cancer, whereas groups C and D contained the highest percentages of stage III and stage IV patients. These results suggest that specific patterns of neutrophil heterogeneity are associated with melanoma progression and may assist in patient grouping and diagnosis.

### Flow cytometry recaptures the seven neutrophil subpopulations in the blood of patients with melanoma

We then asked if the markers highlighted in our in silico analysis could be used to devise a manual gating strategy for replicating the seven neutrophil clusters identified by CyTOF (figure 2A and online supplementary figure 5). Thus, we first used manual gating to isolate total neutrophils from the CyTOF data. These cells overlapped with 90% of our automated CD66b^+^ cell-enriched cluster by backgating (online supplementary figure 5). After applying a manual gate for CD66^+^ neutrophils, we recapitulated the seven subpopulations, by examining the expression patterns of CD117, CD79b, CD45RA, CD16, CD49d, CD101, and CD10. To verify whether manually gated populations mirror automated clusters, we validated this gating strategy by (1) back-gating the manually gated subpopulations to the automated viSNE map and (2) back-gating automated clusters to the manual gates (online supplementary figure 6AB). Both methods confirmed that our manual gating strategy is able to successfully isolate the CyTOF-identified hNeP and Cneut1–Cneut6 neutrophil clusters.

Next, we further validated this manual gating strategy (online supplementary figure 4 and figure 2A) via flow cytometry (online supplementary figure 6C and figure 2B, C). To validate the utility of these findings as they relate to cancer, we investigated neutrophil heterogeneity in five healthy donors (age 23–46, two women, three men) and compared them to five additional treatment-naïve patients with melanoma (aged 59–79 years, two women and three men). We observed that the Cneut2 population comprised >95% of total neutrophils in healthy donors, whereas the other populations (hNeP, Cneut1, Cneut3, Cneut4, Cneut5, and Cneut6) were rarely detected (figure 2C, D). Moreover, compared with healthy donors, these subpopulations (hNeP, Cneut1, Cneut3, Cneut4, Cneut5, and Cneut6) appeared more frequently in treatment-naïve patients with melanoma, which occupied >10% total neutrophils, as determined by flow cytometry. The frequencies of Cneut2, however, were reduced to <90% of total neutrophils in the blood of patients with melanoma. This result is consistent with the CyTOF results we observed in online supplementary figure 2 and prior findings showing that both immature neutrophils and preNeus are mobilized in cancer.^24 25^

### Seven neutrophil subpopulations perform diverse immune functions

Afterwards, we sought to determine whether the seven neutrophil subpopulations we identified express unique immunological phenotypes. Phagocytosis and generation of ROS are two major functions of neutrophils in the immune system.^26^ Therefore, we evaluated the seven neutrophil subpopulations’ phagocytic and ROS-producing capacities.

The seven neutrophil subpopulations from three randomly selected patients with melanoma (age 59, 77, and 79 years, one woman and two men) were analyzed for their phagocytosis of prelabeled zymosan particles. Uptake of zymosan particles by neutrophil subpopulations was then quantified by gating with flow cytometry (red in the zymosan-added (+zymosan) group, figure 3A top panels) and compared with the control (non-zymosan-added (−zymosan) group, figure 3A bottom panels). To determine each subpopulation’s phagocytic ability, we compared the zymosan-positive portion (Zym+Neuts) in each neutrophil subpopulation from the zymosan-added (+zymosan) group. Strikingly, each neutrophil subpopulation performed phagocytosis at different levels of efficiency. Our results show that the hNeP and Cneut1 populations display the highest phagocytic capacities (figure 3B, C). Indeed, the phagocytic abilities of hNeP and Cneut1 were comparable to, or higher than, those of monocytes collected from the same donors (online supplementary figure 7A, B). Moreover, the phagocytic capabilities of the other subpopulations were reduced by 3-fold to >20-fold (figure 3B, C). Despite the fact that Cneut2 and Cneut5 harbored a lower phagocytic capacity compared with hNeP and Cneut1, they were the most prevalent neutrophil subpopulations in the blood of patients with melanoma (figures 1 and 4). Therefore, the absolute numbers of Zym+ Cneut2 and Cneut5 cells still comprise the largest portion (about 50%) of all phagocytic (Zym+) neutrophils and about 25% of total phagocytic CD45^+^ leukocytes (online supplementary figure 7C, D).

Finally, we sought to determine the ability of neutrophil subpopulations to produce ROS. The seven neutrophil subpopulations from three additional randomly selected melanoma-naïve patients (age 59, 77, and 79, one woman and two men) were analyzed by flow cytometry. The ability of gated neutrophils to produce ROS was evaluated in three experimental groups: no stimulation (−sti), ROS inducer (pyocyanin) alone (+sti), or ROS inducer plus ROS inhibitor (+sti+ROSi). We ascertained that all seven neutrophil subpopulations produced ROS on stimulation with pyocyanin and responded to the inhibitor (figure 3D). However, these cells significantly differ from one another in terms of their ability to produce ROS. For instance, the Cneut2 and Cneut5 subpopulations displayed the highest percentage of ROS+ cells in the inducer alone (+sti) group (figure 3E). Meanwhile, populations Cneut4 and Cneut6 showed the lowest ROS-producing capacity of all the subpopulations, with levels comparable to those produced by monocytes (figure 3E and online supplementary figure 7E). To eliminate confounding by non-inducer-specific ROS production by each population during incubation, we compared the fold change (FC) of ROS gMFI in the inducer alone (+sti) group to the no-stimulation (−sti) group. The FC result agreed with our observation that the N_2_ has the highest ROS-producing capacity (figure 3F). Additionally, we found that all subpopulations responded to the ROSi at different levels. In brief, pretreatment with ROSi blocked ROS production in Cneut2 by 85%, whereas the blocking efficacy reached only around 25% in Cneut4 (online supplementary figure 7F). Together, our data indicate that the seven neutrophil subpopulations harbor different immunological capabilities.

## Discussion

Neutrophil heterogeneity has become an active research area, particularly with regard to cancer progression.^18^ Neutrophils carry out contrasting roles in cancer by either killing cancer cells (antitumoral functions) or promoting tumor growth (protumoral functions).^18^ Such observations have long suggested the existence of distinct neutrophil subpopulations. Traditionally, neutrophil subtypes have been separated via gradient centrifugation methods and without use of surface marker characterization, particularly in cancer-related research.^1 18^ The advent of flow cytometry has revealed that neutrophils express a diverse set of surface antigen profiles.^27^ However, due to the limitations imposed by flow cytometry, researchers have had to arbitrarily select only a few surface markers to examine at any particular time. For example, studies in lung cancer research traditionally use one set of markers to identify their neutrophils of interest, whereas studies in infectious diseases use another set of markers; this is also the case for work on the role of neutrophils in both hematopoiesis and angiogenesis.^15 19^ Consequently, neutrophil subpopulations reported by different groups share individual features such as CD66b and/or CD15 but vary in regard to the assessment of other surface antigens. Thus, a lack of consensus markers for neutrophils and their functional subpopulations remains a challenge in the identification of disease-relevant subpopulations.

High-dimensional analysis of neutrophils at the single-cell level is needed to resolve issues associated with identifying neutrophil consensus markers. To meet this demand, we and others have used CyTOF to investigate novel neutrophil progenitors/precursors in BM.^12 13^ Zilionis and colleagues have reported a comprehensive analysis of myeloid cell heterogeneity via single-cell transcriptomics in non-small-cell lung cancer.^17^ This study found six blood neutrophil populations with unique gene signatures in these patients; a myeloid precursor-like population was also found but remained uncharacterized. However, the genes that encode surface markers were not discussed in this study, making it a challenge to compare these neutrophil and myeloid precursors to established neutrophil subpopulations.

Herein, we aimed to establish a paradigm for characterizing neutrophil heterogeneity and correlating specific neutrophil subsets with cancer severity. As such, we designed a CyTOF panel to evaluate the expression of the most commonly used surface antigens in neutrophil samples from patients with melanoma. We identified seven neutrophil clusters in the blood of treatment-naïve patients with melanoma by CyTOF and present evidence indicating that several previously identified neutrophil subpopulations overlap with one another^6 12 16^ and/or represent a mixed pool.^7 8^ This study has also provided a flow cytometry gating strategy, based on our CyTOF work, to recapitulate these seven neutrophil subpopulations. Our results show that these subpopulations display differing capacities to phagocytize debris and produce ROS. Interestingly, a decrease in the ability of T cells to both bind peptide-MHC dimers and respond to specific peptides is induced by myeloid cell-based production of ROS.^28^ Conversely, myeloid production of ROS has been shown to be indispensable for antigen-specific responsiveness in both CD4+ and CD8+ T cells.^29^ This controversial role of ROS in regulating T-cell activity could be partially explained by the dual role of ROS; at low to moderate concentrations, ROS is beneficial for cell survival, whereas high levels of ROS can induce cell death.^30^ Therefore, the abilities of these seven neutrophil subpopulations to produce ROS suggests that they may carry out distinct roles in regulating T-cell responses in cancer. Interestingly, our results show marked similarity between these subpopulations and previously reported neutrophil subtypes with immature developmental statuses. We hypothesize that the heterogeneity we observed in neutrophils derived from patients with cancer may be due to recruitment of preNeus and immature neutrophil populations from the BM to the circulation via cancer-related cytokines such as granulocyte colony-stimulating factor. Because the CyTOF panel used in this study is selective for human blood neutrophil and cancer markers, our results do not capture neutrophil heterogeneity across species, organs, and/or different diseases. Higher dimensions of heterogeneity within our reported subpopulations under such biological conditions should be the focus of future work. Some rare neutrophil subpopulations, such as those expressing T cell receptor (TCR),^31^ could also be lost during our gating. Furthermore, we are unable to rule out the possibility of phenotypic switching between these seven subpopulations and thus consider this notion an interesting direction for follow-up.^6 32^

In summary, our analysis of neutrophil heterogeneity with CyTOF successfully demonstrated marked neutrophil heterogeneity patterns in each treatment-naïve patient sample, based on each cluster’s frequency as a proportion of total neutrophils. We identified groups of patients based on similarities between these neutrophil heterogeneity patterns. The patient groups displayed different melanoma stage categories, suggesting a link between neutrophil heterogeneity and disease, but further validation is needed. We have also shown that these automated clusters can be reproduced by manual gating in conventional flow cytometry. Finally, we demonstrate that these neutrophil subpopulations exhibit significantly different capacities for phagocytosis and ROS production. Thus, we hypothesize these subpopulations play different roles in cancer initiation and/or progression.
