# Supplementary material for: CyTOF mass cytometry reveals phenotypically distinct human blood neutrophil populations differentially correlated with melanoma stage
Source: J Immunother Cancer. 2020 Sep 9;8(2):e000473. doi: 10.1136/jitc-2019-000473 (PMC7482580; doi:10.1136/jitc-2019-000473)
Supplement: Supplementary data [file jitc-2019-000473supp001.pdf]

Table 1. CyTOF antibody panel.

Leukocyte Lineage

Isotope	Metal	Specificity
89	Y	CD45
113	In	CD3
113	In	CD127
115	In	CD41
115	In	CD235a
141	Pr	CD11c
143	Nd	CD123
143	Nd	CD203c
144	Nd	CD19
148	Nd	CD11b
152	Sm	CD66b
163	Dy	CD86
164	Dy	Siglec 8
169	Tm	CD33
176	Yb	CD56

Heterogeneity (other)

Isotope	Metal	Specificity
160	Gd	CD79b

Maturation

Isotope	Metal	Specificity
146	Nd	CD64
151	Eu	CD49d
154	Sm	CD117
156	Gd	CD10
158	Gd	CD101
165	Ho	CD16
166	Er	CD34
167	Er	CD38
172	Yb	CD15

Heterogeneity (adhesion/ activation)

Isotope	Metal	Specificity
142	Nd	CD11a (LFA-1)
145	Nd	CD62L
149	Sm	CD48
155	Gd	CD45RA
170	Er	CD35

Heterogeneity (function)

Isotope	Metal	Specificity
153	Eu	CD14
161	Dy	Arg1
168	Er	CD304 (Nrp1)
171	Yb	HLA-A/B/C
174	Yb	HLA-DR

Heterogeneity (migration)

Isotope	Metal	Specificity
147	Sm	CD182 (CXCR2)
159	Tb	CD197 (CCR7)
175	Lu	CD184 (CXCR4)

Proliferation

Isotope	Metal	Specificity
127	I	IdU
162	Dy	CD71

Table 2. Treatment-Naïve melanoma patient demographics and tumor characteristics.

Characteristics		
Age, y		
	Median (range)	69 (24-82)
Sex, No. (%)		
	Male	12 (57.1)
	Female	9 (42.9)
Primary tumor thickness, mm		
	No. (range)	13 (0.4 - 16)
	0–1.00, No. (%)	4 (19)
	1.00–4.00, No. (%)	6 (28.6)
	>4.00, No. (%)	3 (14.3)
	Missing, No. (%)	8 (38.1)
Primary tumor ulceration status, No. (%)		
	Absent	9 (42.9)
	Present	4 (19)
	Missing	8 (38.1)
Primary tumor anatomic site, No. (%)		
	arms/legs	5 (23.8)
	torso	5 (23.8)
	head/neck	7 (33.3)
	Missing	4 (19)
Primary tumor mitosis, No. (%)		
	Absent	5 (23.8)
	Present	7 (33.3)
	Missing	9 (42.9)
AJCC* stage at pathological diagnosis, No. (%)		
	Stage I	8 (38.1)
	Stage II	4 (19)
	Stage III/IV	5 (23.8)
	Missing	4 (19)

* The American Joint Committee on Cancer (AJCC) Cancer Staging Manual

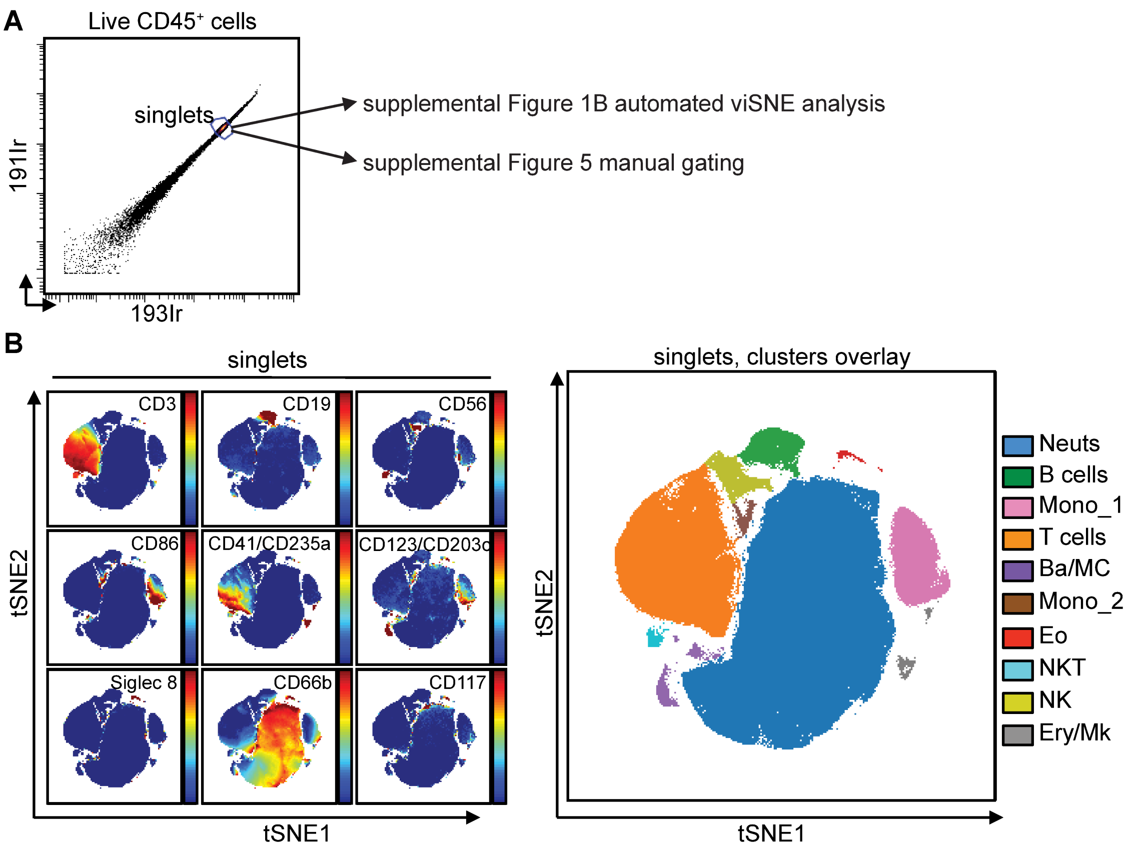

Supplemental Figure 1. Gating strategies to identify the CD66b+ blood neutrophil cluster in melanoma patients with CyTOF.

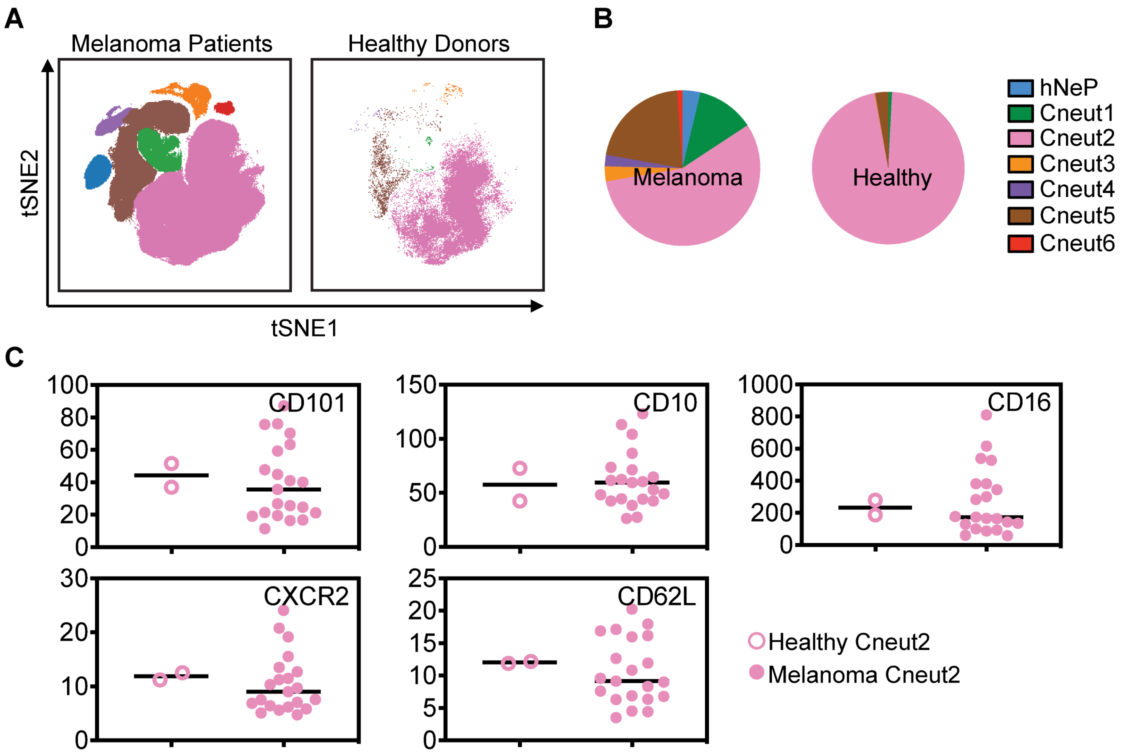

Supplemental Figure 2. Neutrophils display increased heterogeneity in melanoma-patient blood.

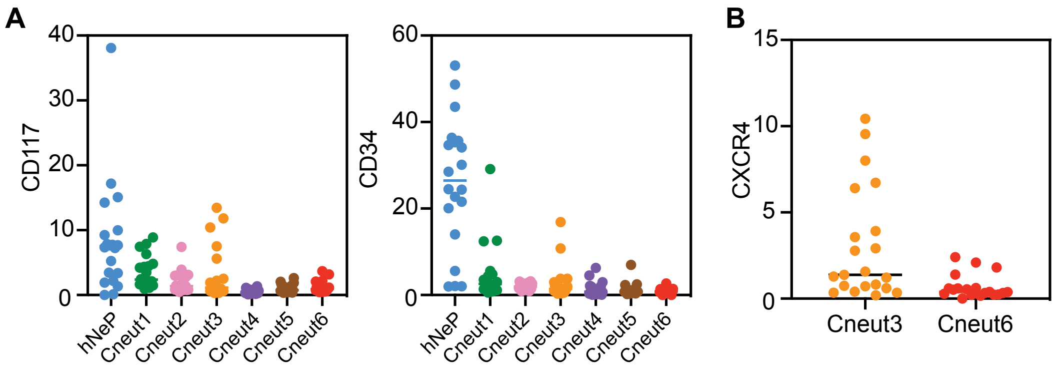

Supplemental Figure 3. Expression levels of markers in 7 automated blood neutrophil clusters.

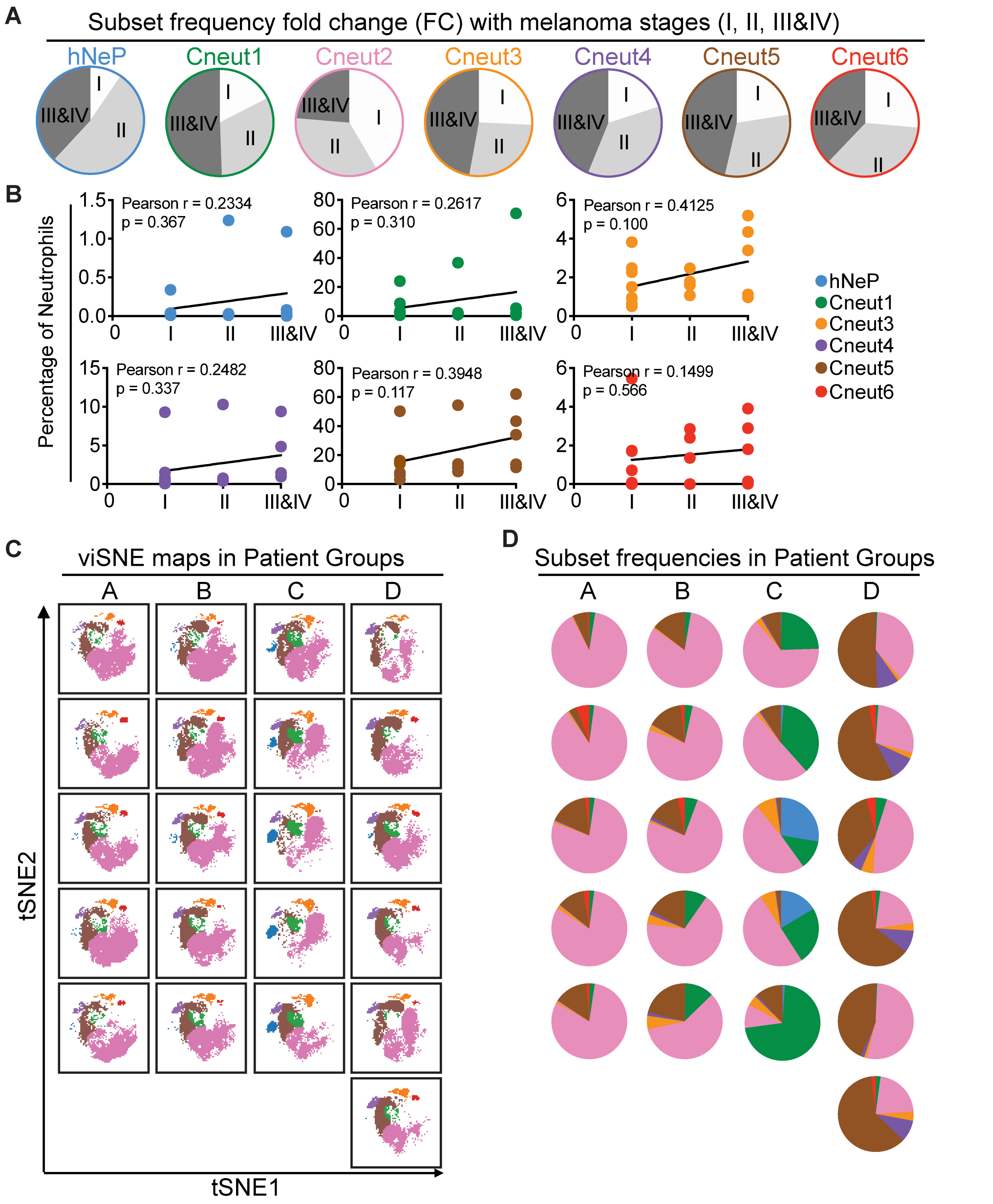

Supplemental Figure 4. FLOWSOM clusters (hNeP and Cneut1-6) shown on viSNE maps categorize melanoma patients into different groups.

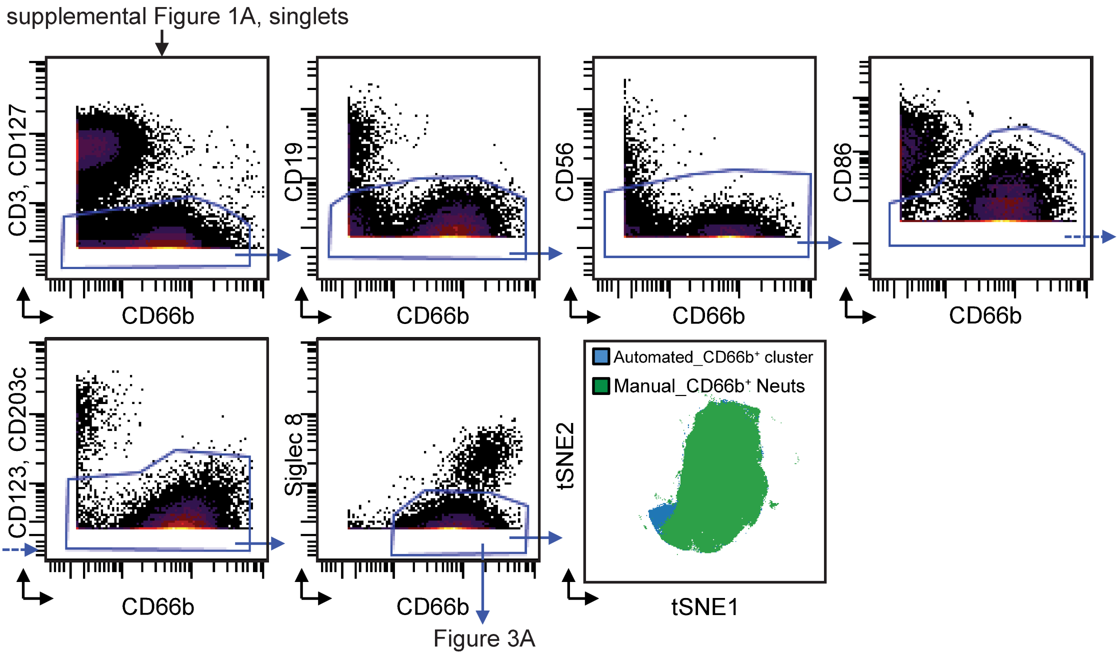

Supplemental Figure 5. Manual gating strategy to select CD66b⁺ blood neutrophils with CyTOF.

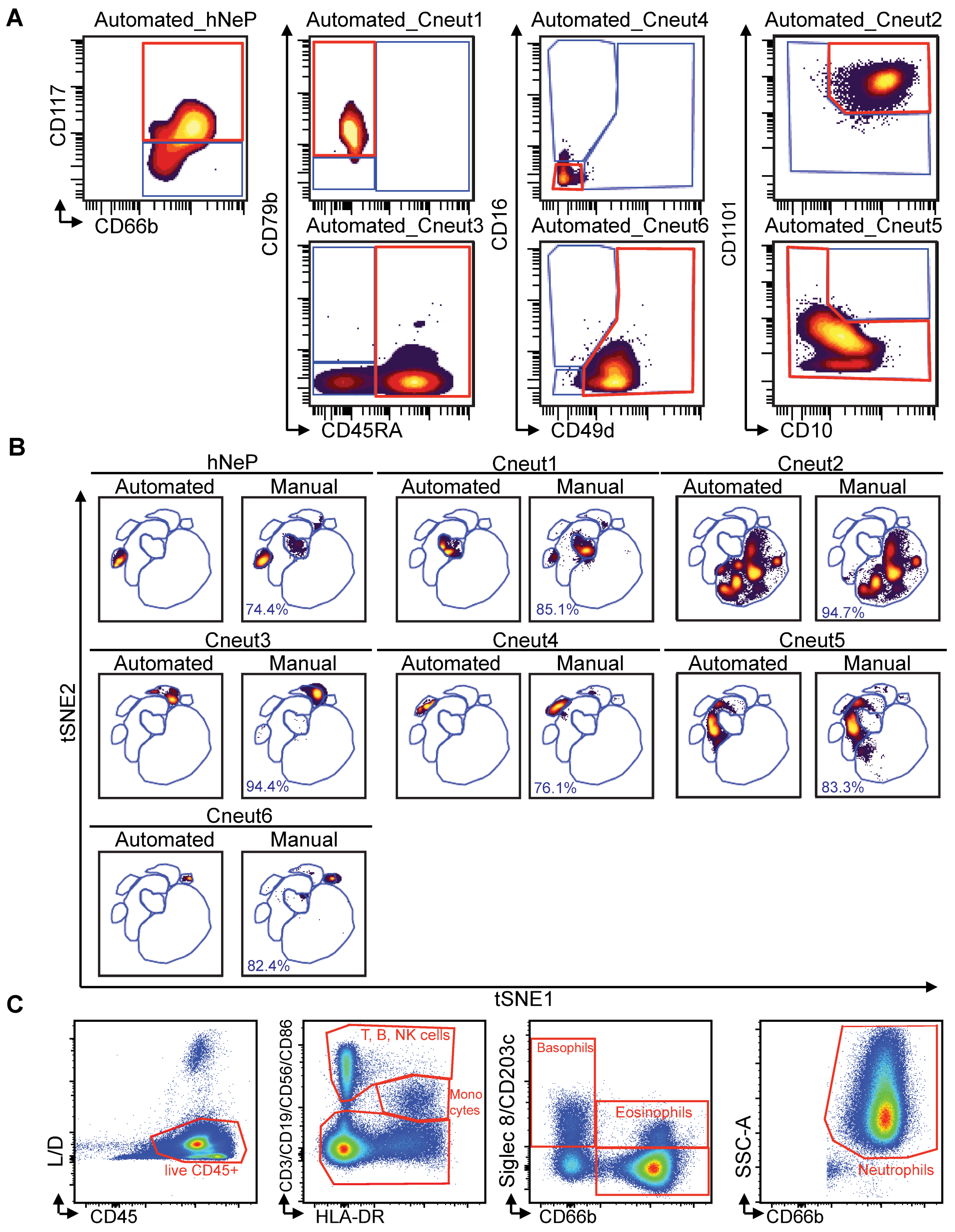

Supplemental Figure 6. Manual gating strategy recapitulates automated neutrophil clusters.

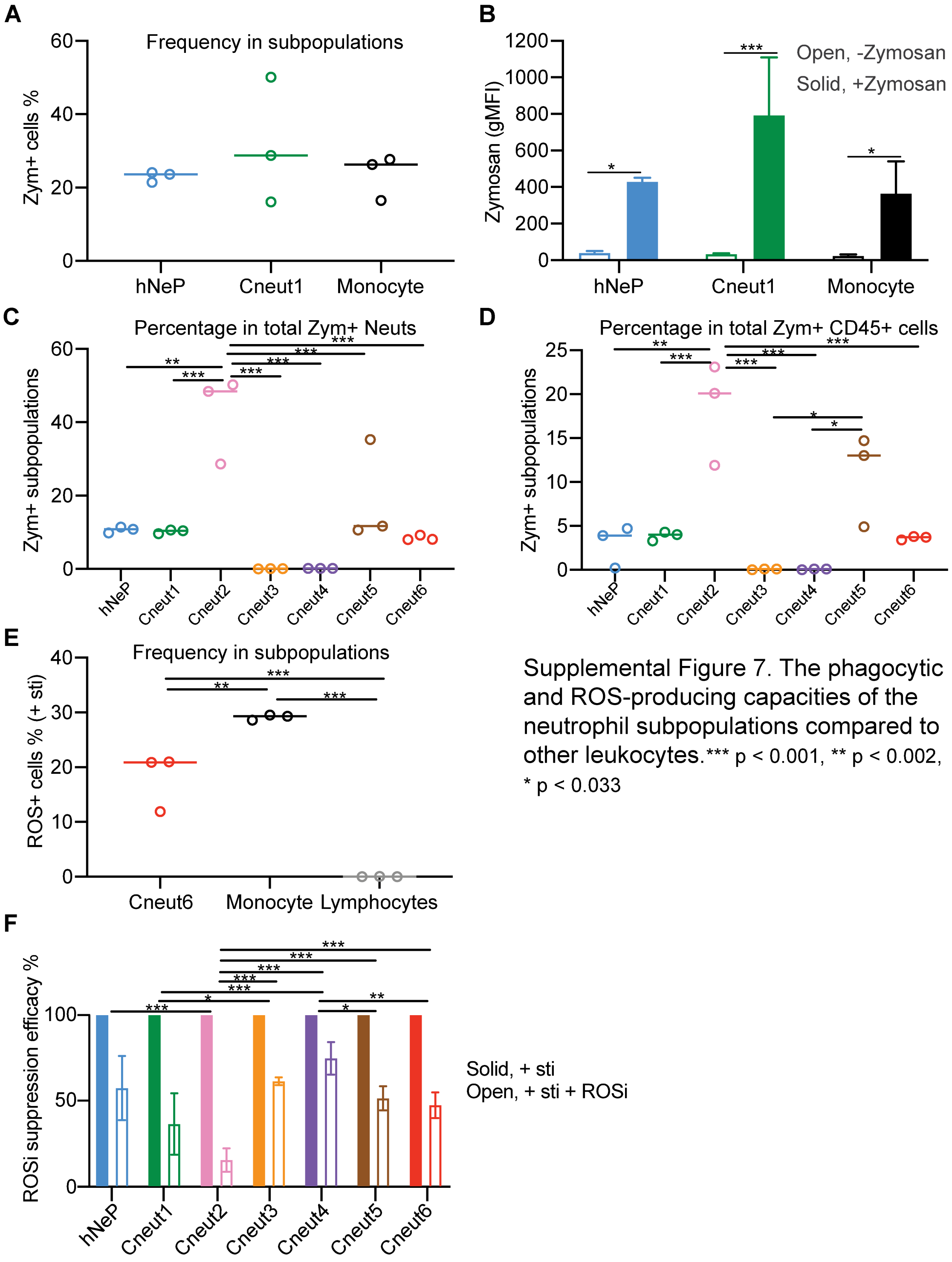

Supplemental Figure 7. The phagocytic and ROS-producing capacities of the neutrophil subpopulations compared to other leukocytes.*** $p < 0.001$, ** $p < 0.002$, * $p < 0.033$
